# The Challenges of Apnea Tests in the Determination of Brain Death in Child Patient on Extracorporeal Membrane Oxygenation

**DOI:** 10.3389/fped.2020.00358

**Published:** 2020-07-07

**Authors:** Lingling Xu, Yujian Liang, Yuan Liao, Jian Rong, Guixing Xu, Wen Tang

**Affiliations:** ^1^Department of Pediatric Intensive Care Unit, The First Affiliated Hospital, Sun Yat-sen University, Guangzhou, China; ^2^Department of Organ Transplantation, The First Affiliated Hospital, Sun Yat-sen University, Guangzhou, China; ^3^Department of Extracorporeal Circulation, The First Affiliated Hospital, Sun Yat-sen University, Guangzhou, China

**Keywords:** apnea test, brain death, ECMO, donation, child

## Abstract

Extracorporeal membrane oxygenation (ECMO) is a life-support modality used in patients with refractory cardiac and/or respiratory failure. ECMO is linked with high risk of neurological complications including brain death. Neurological monitoring during ECMO is important for identifying patients who are suspected of brain death and allows to discontinue ineffective medical treatments. Brain death (BD) is an irreversible cessation of functions of the entire brain, containing the brainstem. The apnea test (AT) is an essential part in the clinical determination of brain death. An apnea test is by neurologic criteria compulsory to confirm BD in China. Apnea test remains a problem for patients receiving ECMO. Currently, there are not any consensus guidelines for the safe performance of AT during ECMO. We report the case of a child on venous-arterial ECMO post-cardiac arrest in whom we performed an apnea test to determine death by neurologic criteria. Decreasing sweep gas flow rate 0.05 L/min every 5 min led to a PaCO_2_ increase of more than 20 mmHg of apnea. The results of the AT was positive. When he was determined brain dead, his parents decided to donate his organs. AT can be performed on potential donor children on ECMO by decreasing the sweep gas flow. It is a safe and effective method and is important for BD determination.

## Introduction

Extracorporeal Membrane Oxygenation (ECMO) is widely used for the treatment of severe acute respiratory failure (Veno-Venous ECMO) or circulatory failure (Veno-Arterial ECMO). ECMO can be established for blood flow, to provide adequate oxygenation and carbon dioxide removal, when support is required for cardiac and/or respiratory failure (1). ECMO is related to a high risk of neurological complications including brain death. In 2016, the national Extracorporeal Life Support Organization registry reported that 10% of 205 children treated with ECMO-cardiopulmonary resuscitation experienced brain death (2). Neurological monitoring during ECMO is important for identifying patients who are suspected of brain death and allows discontinuing ineffective medical treatments. Brain death (BD) is an irreversible cessation of functions of the entire brain. The apnea test is an essential part in the determination of brain death in China. For patients receiving ECMO, the apnea test remains challenging, because vital signs become unstable due to low oxygen saturation and hypotension during the test.

However, there are no consensus guidelines for the safe performance of apnea test (AT) during ECMO, as few literature exists concerning this clinical situation, especially in children (3–6). We reviewed the papers and presented the experience of performing AT on a potential donor supported by ECMO to determine brain death.

## Case Report

A 2-year-old boy, 11 kg, was submerged in a pool for ~5 min on 26 September 2018. Cardiopulmonary resuscitation (CPR) was administered for ~15 min at the scene and during transport to the local hospital. On arrival, he still received cardio-pulmonary resuscitation for about 30 min and his Glasgow coma scale score was three. He demonstrated respiratory failure, heart failure, metabolic acidosis, severe hypotension, and persistent coma. Continuous vasoactive agents (arginine Vasopressin, dopamine, dobutamine, and epinephrine), methylprednisolone, invasive mechanical ventilation, and anti-infection are required to maintain vital signs. However, his vital signs were unstable with low oxygen saturation. He was referred to our hospital for further treatment on 28 September 2018. Clinical examination, Pulse Oxygen Saturation (SPO_2_) was maintained at 90–95% by high level pressure support ventilation. Invasive mechanical ventilation was used with the mode of invasive bi-level positive airway pressure (BIPAP) and the ventilator settings were peak inspiratory airway pressure (PIP) = 26 cmH_2_O; respiratory rate (RR) = 28 breath per minute; positive end-expiratory pressure (PEEP) = 10 cmH_2_O; inspiration time (Ti) = 0.7 s. Mean arterial pressure (MAP) was 60–75 mmHg, while on norepinephrine, 0.8 μg/kg/min, epinephrine, 0.8 μg/kg/min, dopamine, 10 μg/kg/min and dobutamine, 10 μg/kg.min, infusions. He was in deep coma and had no spontaneous breathing without the use of sedatives, analgesics, and muscle relaxant. His Glasgow coma scale score was 3. Pupils were 8 mm and unreactive to a bright light bilaterally. All brain stem reflexes, such as the corneal reflex, pupillary light reflex, vestibulo-ocular reflex, oculocephalogyric reflex, and cough reflex, were absent. The exam was repeated 12 h later and the results remained unchanged.

Laboratory examination at admission: C-reactive protein was 102 mg/L (normal range 0–10), Procalcitonin was 148.10 ng/mL (normal range 0–1), Leukocyte count was 7.10 × 10^9^/L (normal range 4.0–10.0), hemoglobin was 11.0 g/dL (normal range 10.0–12.0), and platelet count was 83 × 10^9^/L (normal range 100–300); blood Urea Nitrogen was 7.4 mmol/L (normal range 2.9–8.6), creatinine was 47 μmol/L (normal, 53–115), Albumin was 34 g/L (normal range 35–50), Total bilirubin was 27.7 μmol/L (normal range 3.0–22.0), Alanine aminotransferase was 190 U/L (normal range 1–40), Aspartate aminotransferase was 205 U/L (normal range 1–37). Echocardiography showed reduced generalized global exercise and left ventricular ejection fraction of 22%.

## Treatment

He was treated with antibiotics, steroids, high doses of vasoactive drugs, mechanical ventilation, and other supportive treatment. He presented with hypotension, tachycardia, and hypoxemia despite ventilatory and vasoactive support. Therefore, after receiving informed the parents' consent, veno-arterial ECMO with low heparinization was performed 10 h after admission using the right carotid and jugular vein cannulation. With ECMO support, his oxygenation was improved, hemodynamic state became stabilized. Organ function was significantly improved and vasoactive drugs were gradually stopped (Table 1). He was still in deep coma without spontaneous breathing. Transcranial doppler showed absence of blood flow signal and electroencephalograph showed electrical silence after 10 h of ECMO therapy for the first time. He was evaluated for brain death because he was suspected of brain death.

**Table 1:** The characteristics of patient before and after received Extracorporeal Membrane Oxygenation.

	**Pre-ECMO**	**10 h Post-ECMO**
**Ventilator parameters (cmH**_**2**_**O)**		
PIP	26	20
PEEP	10	10
FiO_2_ (%)	95	30
**Vasoactive drugs (μg/kg·min)**		
Dopamine	10	2
Dobutamine	10	2
Norepinephrine	0.8	0
Adrenaline	0.8	0
Heart rate (beats/min)	176	143
Blood pressure (mmHg)	89/52	101/83
Urine volume (ml/kg·h)	2.3	4.3
**Arterial Blood Gases (mmHg)**		
PaCO_2_	60	42
PaO_2_	69	86
Blood lactate (mmol/L)	3.6	1.6
ALT (U/L)	190	128
AST (U/L)	205	99
BUN (mmol/L)	7.4	5.2
Scr (μmol/L)	47	57

*PaCO_2_, Partial pressure of carbon dioxide in artery; PaO_2_, Partial arterial oxygen pressure; PIP, Peak inspiratory pressure; PEEP, Positive end expiratory pressure; FiO2, Fraction of inspiration oxygen; ALT, Alanine transaminase; AST, Aspartate Transaminase; BUN, Blood urea nitrogen; Scr, Serum creatinine*.

Criteria for determination of brain death in children in China include four steps (7):

1. The clinical examination confirms the BD diagnose, including deep coma, absence of brainstem reflexes and no spontaneous (SPONT) respiration;
2. Confirmatory tests should fulfill two of the three which include transcranial Doppler (TCD), electroencephalogram (EEG), and somatosensory evoked potential (SEP);
3. The apnea test is positive;
4. A repeat determination should be done 12 h later after the first time.

## The Procedure of Apnea Test in this Case

Precondition of the test: (1) Bladder temperature or rectal temperature ≥35°C (central temperature >35°C). (2) Adjust vasopressors to a systolic blood pressure reach the normal value of the same age group. (3) Preoxygenation with 100% oxygen to an arterial oxygen pressure (PaO_2_) ≥200 mmHg. (4) Arterial partial pressure of carbon dioxide (PaCO_2_) 35–45 mmHg. If PaCO_2_ is less than this level, the gas flow of ECMO should be reduced. If there is chronic hypercapnia, PaCO_2_ may be >45 mmHg.

Steps: (1) Fraction of inspiration oxygen was increased to 100% both on ECMO and ventilator for at least 10 min in order to preoxygenation; (2) reduce gas flow rate 0.05 ml/min of ECMO every 5 min; (3) perform blood gas analysis before reducing gas flow rate; (4) observe SPO_2_ and respiratory movements closely; (5) after finish the test, increase of the gas flow rate and oxygen concentration to improve oxygenation and accelerate CO_2_ clearance.

Determination: If respiratory movements are absent and PaCO_2_ ≥ 60 mmHg or 20 mmHg over a baseline, the test was positive and apnea can be confirmed.

Pitfalls: SPO_2_ < 85% for >30 s, heart rate or blood pressure decrease, arrhythmias, and other signs of deterioration during this examination; AT should be terminated and declared failure. This test needs at least two doctors (one to monitor breathing, blood oxygen saturation, heart rate, cardiac rhythm, and blood pressure; the other to manage the ventilator and ECMO) and one nurse (to manage the oxygen tube and sampling of arterial blood).

## Result

The first apnea test was performed 16 h after ECMO maintenance. Fractional Delivered O_2_ Concentration (FDO_2_) of ECMO was raised to 100% before AT. Ten minutes later, PaO_2_ was 238 mmHg; vital signs remained stable in front, middle, and rear of the test (Table 2). Blood gas analysis was performed and gas flow rate was reduced 0.05 L/min every 5 min. When the gas flow was reduced to 0.15 L/min, PaCO_2_ was increased to 62 mmHg (35 min of AT), no spontaneous respiratory effort was noted. The test was positive. After finishing apnea test, the gas flow rate and oxygen concentration would be temporary increased rather than return to the original settings, as it could improve oxygenation and remove carbon dioxide soon.

**Table 2:** The Extracorporeal Membrane Oxygenation parameters and the child's vital signs before, after, and after 1 h of apnea test.

**ECMO parameter**	**Before apnea test**		**Finish apnea test**		**After apnea test (1 h)**	
	**First**	**Second**	**First**	**Second**	**First**	**Second**
Sweep gas flow rate (L/min)	0.45	0.45	0.15	0.2	0.50	0.60
Pump speed (rpm)	1,797	1,813	1,812	1,826	1,869	1,872
Pump Flow (ml/kg/min)	40	40	40	40	40	40
FiO_2_ (%)	100	100	100	100	60	75
**Arterial Blood Gases**						
PH	7.39	7.42	7.26	7.23	7.35	7.31
PaO_2_ (mmHg)	238	206	78	63	78	65
PaCO_2_ (mmHg)	38	43	62	75	47	52
Blood pressure (mmHg)	101/83	108/76	99/82	96/78	105/85	110/79
Heart rate	143	152	164	168	140	155
SpO_2_ (%)	98	98	92	90	95	94

*ECMO, Extracorporeal Membrane Oxygenation; FiO_2_, Fraction of inspiration oxygen; PaCO_2_, partial pressure of carbon dioxide in artery; PaO_2_, Partial arterial oxygen pressure; SpO2, Pulse Oxygen Saturation*.

According to “Criteria and practical guidance for determination of brain death in children” of China (7), he was first determined brain dead. For children from 1 to 18 years, a repeat assessment should be done 12 h later after the initial evaluation. The second round of EEG and TCD was performed 24 h after ECMO maintenance (14 h after the first EEG) and the second apnea test was performed 32.5 h after ECMO maintenance (16.5 h after the first AT). The apnea test was declared positive when sweep gas flow rate was decreased to 0.2 L/min, PaCO_2_ was increased to 75 mmHg (30 min of AT) as showed in Table 2.

**Figure 1:**
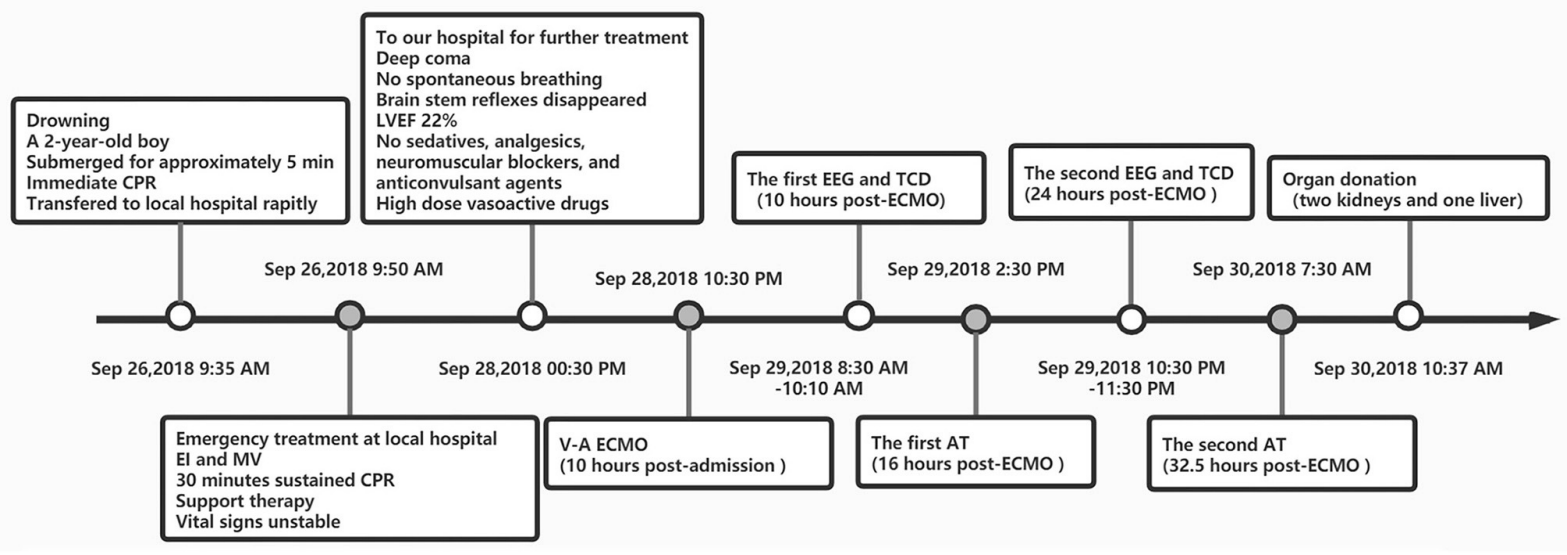
Drowning line. CPR, Cardiopulmonary resuscitation; EI, Endotracheal intubation; MV, mechanical ventilation; LVEF, left ventricular ejection fraction; CPBT, cardiopulmonary bypass team; A-V ECMO, Artero-Venous Extracorporeal membrane oxygenation; ACT, Activated clotting time; AT, Apnea Test; EEG, electroencephalogram; TCD, transcranial doppler sonography; BD, brain death.

He fulfilled all the criteria and was finally determined brain dead. Due to poor prognosis, his parents gave up on further treatment and signed the informed consent to donate the patient's organs. The drowning timeline is shown in Figure 1.

Our patient was transported to the operating room under V-A ECMO support for organ donation. One liver and two kidneys were successfully retrieved for donation. All of the three transplant recipients recovered satisfied after surgery. All of them survived 1.5 years of following up. None of them experienced rejection after organ transplantation.

## Discussion

Brain death is defined as irreversible and concomitant cessation of all cortical, subcortical, and brainstem functions. Despite the general consensus on the concept of BD, its definition still varies widely between countries. All of the clinical evaluation, confirmatory tests, and apnea test must be used to corroborate brain death in China. Confirmatory tests should fulfill two of the three which include TCD, EEG, and SEP (7). However, only one ancillary test should be done to confirm BD when uncertain about the clinical examination or when the apnea test cannot be performed in the United States (8). The strained doctor-patient relationship is the core problem of Chinese medical (9). So the accuracy is the most important thing. Compared with brain death criteria in the United States, Chinese criteria is stricter, lower positive rate, more costly in time, and more reliable by families and doctors. So apnea test is necessary and important for BD determination in China.

The Chinese criteria of BD diagnoses have been officially published in the national journals which represent the legal status of this evaluation (10). The advantage of BD diagnosis is that the families and hospitals can stop unnecessary treatment. The law provides that treatment can be legitimately removed from a patient who was diagnosed BD and the donation of organs for transplantation can start. For our case, his patients decided to donate his organs after he was determined brain dead. So it complies with ethics and related laws and regulations.

In order to diagnose BD, neurological examination should be completed, including apnea testing. Apnea test is an essential component to confirm BD. In patients on ECMO support, however, the AT as described in the American Academy of Neurology practice guidelines is inapplicable (11). The primary goal of AT is to induce hypercapnia to a threshold high enough to stimulate the respiratory drive. It is difficult to perform an apnea test and increase arterial CO_2_ partial pressure (PaCO_2_) during ECMO, because carbon dioxide removal is performed, to a great degree, by the membrane of ECMO. Methods of inducing hypercapnia reflect the factors affecting PaCO_2_ in patients on ECMO, which include decreasing the ECMO sweep flow, adding CO_2_ to the gas mixture, and providing CO_2_ through the ventilator (11). A systematic review reported exposures that 88 patients in 19 studies were evaluated using the AT while on ECMO. Forty-two patients in 14 studies used reducing the sweep flow to induce hypercapnia. Six patients in two studies used providing CO_2_ through the ventilator to induce hypercapnia. One patient used providing CO_2_ through the ECMO oxygenator to induce hypercapnia. Decreasing sweep gas rate allowed reduction in CO_2_ diffusion through the membrane and was the most common change method. However, study reported that one patient developed severe hypoxia because his sweep flow was initially reduced to 0 L/min (12). At present, there is no final conclusion on how appropriate the gas flow should be and what speed to reduce it to be safe, and there are no guidelines for determination of brain death. Some case reports mainly in adults (13–15), but few reports in children.

So we choose this method to perform AT for our patient and did not let the sweep flow decreased to 0 L/min because it could result in hypoxia. Our patient who successfully performed AT test, fulfilled the determination of brain death during VA-ECMO support.

## Conclusion

Determination of brain death, especially the apnea test, remains challenging in children on ECMO. Here, we describe a simple and safe method of apnea testing in a child on ECMO. During the apnea test, reducing the sweep flow by 0.05 L/min every 5 min and increasing the oxygen delivery rate on the ECMO to 100% can significantly increase PaCO_2_, and draw the conclusion that the apnea test is positive without affecting oxygenation.

## Data Availability Statement

All datasets presented in this study are included in the article/supplementary material.

## Ethics Statement

Written informed consent was obtained from the individual(s), and minor(s)' legal guardian/next of kin, for the publication of any potentially identifiable images or data included in this article.

## Author Contributions

WT conceptualized and designed the study, reviewed, and revised the manuscript. LX and YLian carried out the initial analyses and drafted the initial manuscript. LX, YLian, and WT were responsible for the treatment of the patient. GX, YLiao, and JR coordinated and supervised the data collection. YLian and WT critically reviewed the manuscript. All authors read and approved the final manuscript.

## Conflict of Interest

The authors declare that the research was conducted in the absence of any commercial or financial relationships that could be construed as a potential conflict of interest.

## References

[1] Betit P. Technical advances in the field of ECMO. Respir Care. (2018) 63:1162–73. 10.4187/respcare.06320 30166411

[2] Barbaro RP Paden ML Guner YS Raman L Ryerson LM Alexander P Pediatric extracorporeal life support organization registry international report 2016. ASAIO J. (2017) 63:456–63. 10.1097/MAT.0000000000000603 28557863 PMC5626007

[3] Jarrah RJ Ajizian SJ Agarwal S Copus SC Nakagawa TA. Developing a standard method for apnea testing in the determination of brain death for patients on venoarterial extracorporeal membrane oxygenation: a pediatric case series. Pediatr Crit Care Med. (2014) 15:e38–43. 10.1097/PCC.0000000000000006 24201855

[4] Shah V Lazaridis C. Apnea testing on extracorporeal membrane oxygenation: case report and literature review. J Crit Care. (2015) 30:784–6. 10.1016/j.jcrc.2015.03.028 25891646

[5] Smilevitch P Lonjaret L Fourcade O Geeraerts T. Apnea test for brain death determination in a patient on extracorporeal membrane oxygenation. Neurocrit Care. (2013) 19:215–7. 10.1007/s12028-013-9845-y 23615865

[6] Harrar DB Kukreti V Dean NP Berger JR Carpenter JL. Clinical determination of brain death in children supported by extracorporeal membrane oxygenation. Neurocrit Care. (2019) 31:304–11. 10.1007/s12028-019-00700-z 30891693

[7] Brain Injury Evaluation Quality Control Centre of National Health and Family Planning Commission. Criteria and practical guidance for determination of brain death in children (BQCC version). Chin Med J (Engl). (2014) 127:4140–4. 25430464

[8] Wijdicks EF Varelas PN Gronseth GS Greer DM Evidence-based guideline update: determining brain death in adults: report of the Quality Standards Subcommittee of the American Academy of Neurology. Neurology. (2010) 74:1911–8. 10.1212/WNL.0b013e3181e242a8 20530327

[9] Liu J Miao J Zhang D. Dilemma of healthcare reform and invention of new discipline of health fiscalogy. Glob Health Res Policy. (2016) 1:4. 10.1186/s41256-016-0003-x 29202054 PMC5675063

[10] Ding ZY Zhang Q Wu JW Yang ZH Zhao XQ A comparison of brain death criteria between China and the United States. Chin Med J (Engl). (2015) 128:2896–901. 10.4103/0366-6999.168047 26521787 PMC4756902

[11] Migdady I Stephens RS Price C Geocadin RG Whitman G Cho SM. The use of apnea test and brain death determination in patients on extracorporeal membrane oxygenation: a systematic review. J Thorac Cardiovasc Surg. (2020) 21:S0022–5223. 10.1016/j.jtcvs.2020.03.038 32312535

[12] Yang HY Lin CY Tsai YT Lee CY Tsai CS. Experience of heart transplantation from hemodynamically unstable brain-dead donors with extracorporeal support. Clin Transplant. (2012) 26:792–6. 10.1111/j.1399-0012.2011.01585.x 22280372

[13] Muralidharan R Mateen FJ Shinohara RT Schears GJ Wijdicks EF. The challenges with brain death determination in adult patients on extracorporeal membrane oxygenation. Neurocritcare. (2011) 14:423–6. 10.1007/s12028-011-9516-9 21327575 PMC7816482

[14] Madden M Andrews P Rector R Menaker J Habashi N. Carbogen for apnea testing during the brain death declaration process in subjects on extracorporeal membrane oxygenation. Respir Care. (2020) 65:75–81. 10.4187/respcare.06378 31690613

[15] Giani M Scaravilli V Colombo SM Confalonieri A Leo R Maggioni E. Apnea test during brain death assessment in mechanically ventilated and ECMO patients. Intensive Care Med. (2016) 42:72–81. 10.1007/s00134-015-4105-6 26556611

